# Pharmacokinetics and Toxicity Overview of Active Compounds Berberine, Palmatine, and Jatrorrhizine From *Fibraurea tinctoria* Lour: Drug‐Likeness, ADMET Prediction, and In Vivo Extract Toxicity Assessment

**DOI:** 10.1155/jt/7251602

**Published:** 2025-12-11

**Authors:** Indah Purwaningsih, Iman Permana Maksum, Dadan Sumiarsa, Sriwidodo Sriwidodo

**Affiliations:** ^1^ Department of Chemistry, Faculty of Mathematics and Natural Science, Universitas Padjadjaran, Sumedang, 45363, Indonesia, unpad.ac.id; ^2^ Department of Medical Laboratory Technology, Poltekkes Kemenkes Pontianak, Pontianak, 78124, Indonesia, poltekkes-pontianak.ac.id; ^3^ Department of Pharmaceutics and Pharmaceutical Technology, Faculty of Pharmacy, Universitas Padjadjaran, Sumedang, 45363, Indonesia, unpad.ac.id

## Abstract

*Fibraurea tinctoria* Lour has long been used by the indigenous ethnic groups of Kalimantan in the traditional treatment of malaria, jaundice, and diabetes mellitus. This study aimed to evaluate the drug‐likeness and ADMET properties of the active compounds berberine, palmatine, and jatrorrhizine and to assess the acute toxicity of the plant extract using in silico and in vivo approaches. In silico analysis was performed using the pkCSM, ProTox‐II, and SwissADME online web servers to predict drug‐likeness and ADMET of compounds from *Fibraurea tinctoria* Lour. The in vivo acute toxicity of the extract was evaluated according to the Organization for Economic Cooperation and Development (OECD) 425 guideline. In silico studies have demonstrated that berberine, palmatine, and jatrorrhizine exhibit favorable drug‐likeness and pharmacokinetic properties but indicate potential oral toxicity. In contrast, the in vivo acute toxicity study revealed no toxicity or adverse effects, with an LD_50_ greater than 5000 mg/kg. These findings indicate that despite the in silico prediction showing the potential toxicity of these three compounds, the extract exhibited relative safety based on in vivo tests and has the potential for further pharmacological development.

## 1. Introduction

Natural product‐based​ traditional medicine has an extensive historical background and is widely used in numerous countries for health maintenance, disease prevention, and treatment of diverse medical conditions. Ancestral healing practices are frequently transmitted through familial lineages as components of cultural traditions. Consequently, individuals often use these remedies without considering potential adverse effects [1].

*Fibraurea tinctoria* has long been used by indigenous tribes in Kalimantan for the treatment of various diseases, including malaria, jaundice, and diabetes mellitus. The bark, leaves, roots, and stems were the most frequently used sections. This plant exhibits many pharmacological activities, including antidiabetic, antioxidant, anti‐inflammatory, antimalarial, antimicrobial, and anticancer activities [2–4]. In vivo studies have shown that this plant extract effectively reduces blood glucose levels in animals with diabetes mellitus [5–7]. Evaluation of antioxidant activity using DPPH and mSOD methods showed that this plant has good antioxidant activity [3]. Phytochemical analysis of this plant has revealed the presence of secondary metabolites, including alkaloids, phenolics, flavonoids, saponins, tannins, steroids, and triterpenoids [8].

**Figure 1:**
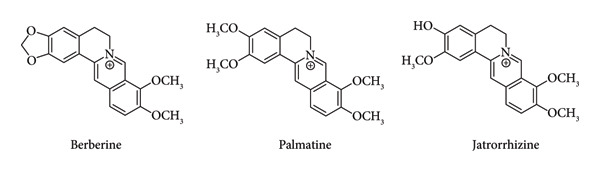
Chemical structure of berberine, palmatine, and jatrorrhizine.

Berberine, palmatine, and jatrorrhizine are quaternary ammonium salts of the protoberberine group in the isoquinoline alkaloids. These compounds are found in *Fibraurea tinctoria* and exhibit antidiabetic activities [2, 3, 9–11]. The molecular structures of these three compounds exhibit close structural relationships, with only minor variations in the substitution pattern of the isoquinoline moiety [12] (Figure 1). Berberine possesses a methylenedioxy group at the C2 and C3 positions of its tetracyclic framework. Jatrorrhizine contains three methoxyl groups and one hydroxy group, whereas palmatine contains​ four methoxyl groups [2].

Currently, in silico methods have been widely applied in various fields, particularly drug development, as initial screening tools to identify potentially new compounds. These methods play an important role in drug design and discovery by efficiently predicting drug efficacy and safety using computational models [13, 14]. In silico models that enable the early estimation of several ADMET properties have been developed through a comprehensive understanding of the links between significant ADMET parameters, molecular structure, and properties. Studies have shown that poor pharmacokinetic profiles, including lack of clinical efficacy (40%–50%), uncontrolled toxicity (30%), and inadequate drug‐like properties (10%–15%), have been responsible for nearly 90% of medication failures over the last decade. Therefore, early pharmacokinetic properties and toxicity prediction are critical for drug development to avoid costly and unnecessary failures [15].

It is a common misperception that natural products and herbal remedies are risk‐free and have no negative side effects compared to synthetic drugs. Certain phytochemical substances can interact with biological systems in various ways after administration. These interactions can have unfavorable and occasionally lethal effects [16]. Therefore, toxicological evaluations are required to ensure the safety of newly developed drugs and herbal constituents.

Although the pharmacological activities of berberine and other protoberberine alkaloids, including palmatine and jatrorrhizine, have been widely documented, scientific studies specifically focusing on *Fibraurea tinctoria* Lour remain limited. Based on our comprehensive review of the published literature, we did not identify any extensive studies investigating the toxicological properties of *Fibraurea tinctoria* Lour stem extract. This study aimed to fill this gap by combining in silico and in vivo approaches. Specifically, we examined the drug‐likeness and ADMET properties of the active compounds berberine, palmatine, and jatrorrhizine using in silico studies and further assessed the acute toxicity of the extracts through in vivo experimentation. The integration of pharmacokinetic and toxicity prediction with experimental validation provides a novel and more comprehensive safety profile of *Fibraurea tinctoria* Lour, offering important insights that have not been previously reported and strengthening the scientific basis for its potential therapeutic applications.

## 2. Materials and Methods

### 2.1. Materials

Botanical specimens were collected in August 2021 from Menua Sadap Village in Indonesia’s West Kalimantan Province. The samples were subsequently authenticated by the Department of Biology of Tanjung Pura University, Faculty of Mathematics and Natural Science (No. 119/A/LB/FMIPA/UNTAN/2021). Ethanol 96% was used for extraction, and sodium carboxymethyl cellulose (CMC‐Na) was used as the suspending agent for the extract. For the in silico studies, three‐dimensional molecular structures of berberine, palmatine, and jatrorrhizine were obtained from PubChem (https://pubchem.ncbi.nlm.nih.gov/).

### 2.2. Methods

#### 2.2.1. Drug‐Likeness

This study used an online web server (http://www.swissadme.ch/) to analyze drug‐likeness properties, including Lipinski’s Rule of Five (LO5) and Veber’s rule.

#### 2.2.2. ADMET Prediction

The online web server pkCSM (https://biosig.lab.uq.edu.au/pkcsm/prediction) was used to predict pharmacokinetic characteristics, such as absorption, distribution, metabolism, and excretion. Toxicity predictions were obtained using a web server (https://tox-new.charite.de/protox_II/index.php).

#### 2.2.3. Acute Oral Toxicity

##### 2.2.3.1. Preparation of Extract

Prior to extraction, all dried samples were pulverized and sieved through a 40‐mesh sieve. Air‐dried and powdered stems (1.00 kg) were extracted using 96% ethanol at room temperature (4 × 4 L, 24 h each). The extract was concentrated by solvent evaporation, yielding an ethanol extract weighing 26.3 g. Subsequently, 2000 and 5000 mg/kg test doses were prepared using 0.5% CMC‐Na as a suspending agent.

##### 2.2.3.2. Ethical Clearance

This study was conducted at the Pharmacology and Toxicology Laboratory of the Faculty of Pharmacy, Padjadjaran University. The code of ethics for study was approved by the Research Ethics Commission of Padjadjaran University (No. 729/UN6.KEP/EC/2024).

##### 2.2.3.3. Selection and Maintenance of Animals

The animals used in this study were ddY strain mice (*Mus musculus*). Healthy nulliparous and nonpregnant female mice, aged 8–12 weeks, with variations in body weight not exceeding 20% of the average body weight, were randomly selected. All experimental animals were maintained under standardized environmental conditions (room temperature 22 ± 3°C, relative humidity 30%–70%, and 12 h light/dark cycle). The mice underwent a 7‐day acclimatization period to adapt to the laboratory conditions. During acclimatization, the mice were provided with food and water ad libitum.

##### 2.2.3.4. Acute Oral Toxicity Procedure

The acute oral toxicity test of *Fibraurea tinctoria* Lour extract was conducted according to the OECD 425 Guideline: Up‐and‐Down Procedure (UDP), which consists of a limit and a main test. The limit test is a sequential test that uses a maximum of five animals with test doses of 2000 or 5000 mg/kg. The main test was performed to determine if mortality occurred during the limit test. In the limit test 2000, the first animal was administered a dose of 2000 mg/kg. If mortality occurred, the main test was performed to determine the LD_50_. However, if the animal survived after 24 h of observation, the same dose was administered to additional animals until a maximum of five animals were tested. When three or more animals survived, the LD_50_ was considered to be greater than 2000 mg/kg, and testing proceeded to the limit test 5000. In the limit test 5000, if three or more animals survived, the LD_50_ was concluded to be greater than 5000 mg/kg. Conversely, if three or more animals died, the main test was performed using the highest dose (5000 mg/kg).

The mice were kept without food for 3–4 h before dosing but had access to water *ad libitum*. The body weight of the mice was measured, and the dose was administered based on their body weight. The extract suspension was freshly prepared prior to administration. Food was provided 1–2 h after dosing. The mice were observed for changes in behavioral or toxicity symptoms for the first 30 min, and then for 1, 2, 4, and 24 h after dosing, and the observations continued for 14 days. The control group (*n* = 5) was administered 0.5% CMC‐Na. The body weights of the mice were recorded throughout the study (14 days). On Day 15, all mice were sacrificed by cervical dislocation, and vital organs, including the heart, kidneys, liver, lungs, and spleen, were collected. The absolute and relative weights of the organs were determined. Absolute organ weights were obtained by directly weighing the organs, and relative organ weights were calculated as the ratio of absolute organ weight to body weight (g/g) and expressed as percentages. The LD_50_ was calculated based on the maximum likelihood method using AOT425StatPgm.

##### 2.2.3.5. Statistical Analysis

Experimental data are presented as mean ± standard error of the mean (SEM). Statistical analyses were performed using SPSS (Version 23). One‐way ANOVA was applied to evaluate the relative organ weight data when the normality assumption was met. Conversely, for parameters that did not meet the assumption, the Kruskal–Wallis test was used, followed by the Bonferroni post hoc test. Differences were considered statistically significant at *p* < 0.05.

## 3. Results

### 3.1. Drug‐Likeness

In the present study, drug‐likeness of the compounds was predicted using LO5 and Veber’s rule. According to Lipinski, an active compound should comply with all LO5 parameters, with no more than one violation. The required parameters include molecular weight ≤ 500 Da, hydrogen bond donor ≤ 5, hydrogen bond acceptor ≤ 10, log *P* ≤ 5, and molar refractivity between 40 and 130 [17]. Meanwhile, in Veber’s rule, the measured parameters are the topology polar surface area (TPSA) and the number of rotatable bonds (nRotb). A compound is considered to have good bioavailability if TPSA ≤ 140 Å^2^, whereas a rotatable bond number greater than 10 indicates low oral bioavailability [18, 19]. The drug‐likeness analysis according to Lipinski’s and Veber’s rules is shown in Table 1.

**Table 1:** The drug‐likeness analysis for Lipinski’s and Veber’s rules.

Rule	Parameters	Ligand		
		Berberine	Palmatine	Jatrorrhizine
Lipinski’s Rule of Five	Molecular mass	336.36	352.4	338.38
	Hydrogen bond donor	0	0	1
	Hydrogen bond acceptors	4	4	4
	LogP	3.10	3.38	3.08
	Molar refractivity	94.87	101.8	97.33
	Violation	0	0	0
	Drug‐likeness	Yes	Yes	Yes

Veber’s rule	Total polar surface area	40.80	40.80	51.80
	Total number of rotatable bonds	2	4	3

### 3.2. ADMET Prediction

This study assessed the ADMET profiles of berberine, palmatine, and jatrorrhizine. The absorption parameters were evaluated by examining water solubility and intestinal absorption. Distribution was analyzed using three parameters: volume of distribution (VDss), blood–brain barrier (BBB) permeability, and central nervous system (CNS) permeability. Metabolism parameters were evaluated by predicting the probability of compounds acting as substrates or inhibitors of cytochrome P450 (CYP) enzymes. The excretion parameter was determined by measuring the total clearance, and toxicity was assessed using the lethal dose 50 (LD_50_) value. The overall prediction results are presented in Table 2.

**Table 2:** The ADMET analysis.

Properties	Parameters		Ligands		
			Berberine	Palmatine	Jatrorrhizine
Absorption	Water solubility (log mol/L)		−3.97	−4.194	−3.871
	Intestinal absorption (% Absorbed)		97.147	97.084	94.465

Distribution	Volume of distribution/VDss (log L/kg)		0.580	0.641	0.539
	BBB permeability (log BB)		0.198	−0.112	−0.150
	CNS permeability (log PS)		−1.543	−1.535	−2.142

Metabolism	Inhibitors of	CYP1A2	Yes	Yes	Yes
		CYP2C19	No	No	No
		CYP2C9	No	No	No
		CYP2D6	Yes	Yes	Yes
		CYP3A4	Yes	No	No
	Substrate of	CYP2D6	No	No	No
		CYP3A4	Yes	Yes	Yes

Excretion	Total clearance (log mL/min/kg)		1.270	1.246	1.222
Toxicity	Lethal dose 50/LD_50_ (mg/kg)		200	200	200

### 3.3. Acute Oral Toxicity

#### 3.3.1. Behavioral Parameters and Mortality

All groups were observed for the first 30 min, followed by 1, 2, 4, and 24 h after dosing, and continued for 14 days to identify signs and toxicity symptoms or behavioral changes. The behavioral parameters observed included motor activity, gesture, hanging, retablismen retablissement reflex (righting reflex), respiration, vocalization, piloerection, grooming, straub, tremor, writhing, convulsions, catalepsy, flexion, haffner, ptosis, lacrimation, corneal reflex, pineal reflex, salivation, defecation, urination, and mortality. Behavioral monitoring in limit tests 2000 and 5000 revealed no behavioral changes or toxic symptoms in any of the parameters compared to the control group, confirming the safety of acute administration of the extract. Furthermore, no mortality was recorded in the limit tests 2000 and 5000 during the 14‐day observation period. Analysis using the AOT425StatPgm program concluded that the LD_50_ of the ethanol extract of *Fibraurea tinctoria* Lour stem was greater than 5000 mg/kg.

#### 3.3.2. Body Weight

**Figure 2:**
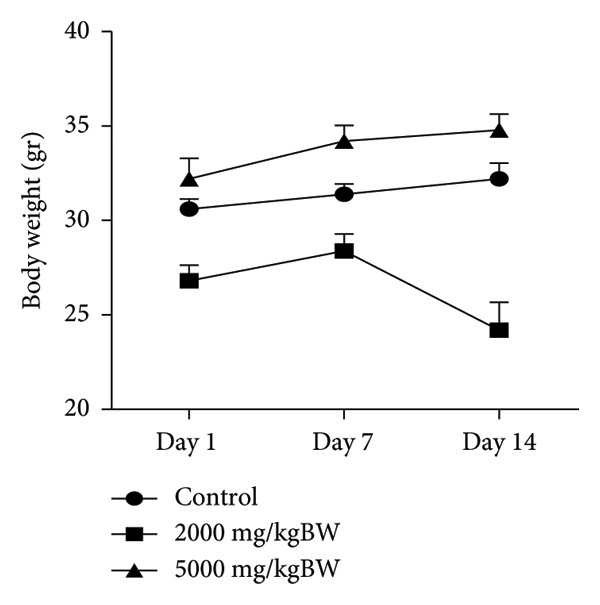
Effect of extract administration on body weight.

The analysis results indicated that the administration of the extract led to an increase in body weight in all experimental groups on Day 7 compared to Day 1. However, only the control and 5000 mg/kg dose groups exhibited an increase in body weight on Day 14 compared with Day 1, whereas the 2000 mg/kg dose group exhibited a decrease in body weight. The results are shown in Figure 2.

#### 3.3.3. Relative Organ Weight

**Figure 3:**
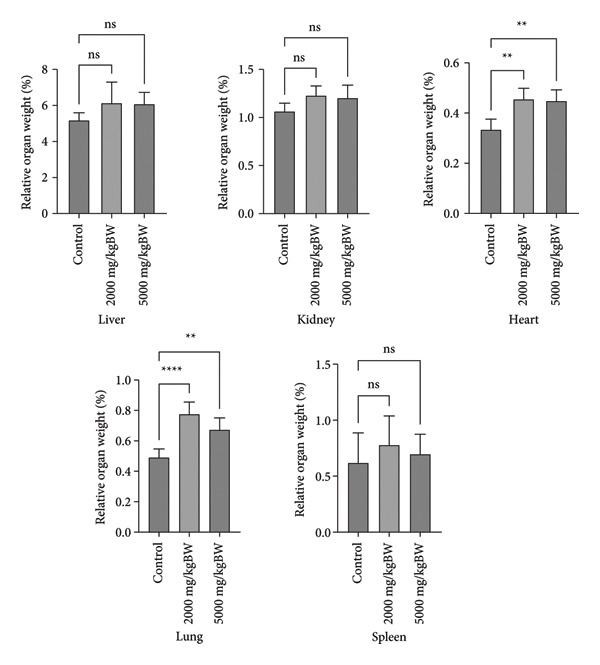
Effect of extract administration on relative organ weight.

There were no significant differences in the relative organ weights of the liver, kidneys, and spleen among the 2000 and 5000 mg/kg dose groups compared to the control group (*p* > 0.05). In contrast, the heart and lungs showed significant differences between the 2000 and 5000 mg/kg dose groups and the control group (*p* < 0.05). The results are shown in Figure 3.

## 4. Discussions

### 4.1. Drug‐Likeness

Active compounds with potential medicinal uses must comply with LO5. This rule determines whether a chemical compound with certain pharmacological or biological activities has physicochemical properties that render it active orally. Lipinski’s rule is a computational procedure used to estimate acceptable water and lipid solubility and permeability of compounds in the gastrointestinal tract. Thus, it can be used as the initial step in determining the oral bioavailability of an active compound [20]. LO5 includes molecular weight, hydrogen bond donor, hydrogen bond acceptor, log P, and molar refractivity. These parameters are related to 90% of the activity of the orally administered drugs. If an active compound does not meet Lipinski’s rules, it will most likely face activity problems in the future, although this does not guarantee that compounds that meet these rules will not have activity problems [17]. In addition to Lipinski’s rule, Veber’s rule is commonly used to evaluate the oral bioavailability of compounds. This rule complements Lipinski’s rule by providing additional parameters for predicting drug‐likeness. According to Veber’s rule, the parameters tested were the TPSA and the nRotb. Compounds become more flexible and can interact with the target receptor when the TPSA is less than 140 Å^2^ and the nRotb is less than 10 [21].

The results showed that berberine, palmatine, and jatrorrhizine complied with the criteria of Lipinski’s and Veber’s rules. The fulfillment of these two rules indicates that these three compounds have physicochemical profiles that support their oral bioavailability. This suggests that these compounds have the potential to be well absorbed through the gastrointestinal tract, have adequate solubility and permeability, and thus increase the possibility of reaching their biological targets in vivo. Nevertheless, although these in silico findings support their pharmacological potential, compliance with these rules does not guarantee good in vivo bioavailability of the compounds. Additional factors, such as metabolism, plasma protein binding, and excretion pathways, influence pharmacokinetic profiles. Therefore, these results serve as an initial basis for further research through in vivo pharmacokinetic and biological activity studies of these compounds.

### 4.2. ADMET Prediction

Two important parameters were measured to predict absorption: water solubility and human intestinal absorption (HIA). Berberine, palmatine, and jatrorrhizine were predicted to have low water solubility, suggesting low bioavailability and the requirement of high doses to achieve therapeutic concentrations after oral administration [22]. In contrast, the predicted HIA values showed that these three compounds exhibited good absorption, with absorption rates exceeding 90%. HIA is an important step in transporting compounds to desired targets [23]. High HIA values (> 90%) indicate that these three compounds have the potential to be well absorbed by the human intestine. Therefore, the main obstacle lies in the solubility and not in the absorption capacity of the intestinal membranes.

VDss indicates the extent of drug distribution in body fluids and tissues and is strongly related to plasma protein binding [24]. All three compounds were predicted to have large distribution volumes (log VDss > 0.45), with palmatine showing the highest value, suggesting a broader tissue distribution than berberine and jatrorrhizine. Regarding BBB and CNS parameters, both palmatine and jatrorrhizine exhibited lower BBB permeability (log BB < −1) than berberine (log BB > −1), and all three compounds demonstrated the capacity to readily penetrate the CNS (log PS > −2). BBB and CNS are important parameters that must be considered when evaluating the efficacy of drugs targeting the brain or reducing unwanted side effects and toxicity [25–27].

CYP is an important enzyme involved in drug metabolism. Five specific CYP enzymes (CYP2D6, CYP2C9, CYP1A2, CYP3A4, and CYP2C19) were used to analyze the metabolic processes of the lead compounds in this study. Among these, CYP3A4 is particularly significant as one of the most important members of the metabolic enzyme family, as it is responsible for metabolizing approximately half of the medications currently available [28]. The results showed that all three compounds were substrates of CYP3A4, indicating that their metabolism was mediated by this enzyme and may be affected by competition with other drugs metabolized via the same pathway. Conversely, these three compounds act as inhibitors of CYP1A2 and CYP2D6; however, for CYP3A4, only berberine inhibits this isoform. Inhibitors have indicated that these compounds can suppress metabolic activity and lead to unwanted effects in the body. Inhibition of CYP enzymes is known to significantly influence the pharmacokinetics of a compound and is one of the main causes of pharmacokinetic‐related drug interactions that cause toxic side effects or unwanted drug reactions owing to the lower clearance and accumulation of the drug or its metabolites in the body [29]. Therefore, evaluating the ability of a compound to inhibit this enzyme is important to assess its safety and interactions with other compounds.

Total clearance is a critical pharmacokinetic parameter that facilitates the estimation of the required dose and provides the compound exposure level (AUC) necessary for a therapeutic effect. A higher clearance value reflects faster excretion, whereas a lower clearance value indicates a longer persistence of the compound in the body [30]. Berberine exhibited a higher total clearance than palmatine and jatrorrhizine, although the difference was not statistically significant. This finding suggests that berberine is eliminated more rapidly than the other two compounds and requires more frequent dosing intervals to maintain therapeutic levels.

The final parameter examined in the ADMET profiles in this study was toxicity. In silico toxicology prediction is an emerging and valuable approach that reduces the reliance on laboratory animals, which are time‐consuming and expensive. In toxicity testing, the toxic dose of a compound is expressed as the LD_50_ value, defined as the dose of a substance that causes death in 50% of the test population, typically reported as mg/kg body weight [31, 32].

In silico toxicity prediction showed that the LD_50_ of berberine, palmatine, and jatrorrhizine were 200 mg/kg. Therefore, they were categorized as toxic if ingested (50 < LD_50_ ≤ 300). This prediction provides an initial overview of the need to monitor these three compounds from a safety perspective. However, these results differ significantly from in vivo acute toxicity tests, where the LD_50_ values for berberine and palmatine were reported to be 713.57 and 1533.68 mg/kg, respectively [33], and jatrorrhizine reached 5500 mg/kg [34]. These results indicate that in silico toxicity predictions tend to provide higher toxicity estimates than in vivo assessments. The discrepancy between the in silico and in vivo results reflects the limitations of the computational prediction models.

Complementary in vitro studies have provided further insights into the cytotoxicity at the cellular level. The in vitro test results showed that cytotoxic activity varied between the compounds and cell types. Jatrorrhizine exhibited cytotoxic effects in SW480 human colon cancer cells (200 μg/mL) and HepG2 hepatocellular carcinoma cells (100 μM). Slight cytotoxicity was reported in MCF10A normal breast epithelial cells at 100 μM, but no cytotoxicity was observed in MCF‐7 cells at concentrations below 10 μM [9]. Palmatine exhibited dose‐dependent cytotoxic effects on human estrogen receptor‐positive breast cancer cell lines (MCF7, T47D, and ZR‐75‐1). Palmatine reduced cell viability and proliferation, with IC_50_ values ranging from 5.126 to 5.805 μg/mL. Importantly, palmatine exhibited lower cytotoxicity toward normal human breast epithelial cells (MCF‐10A), indicating its selectivity for cancer cells. Cytotoxicity is associated with apoptosis induction, as evidenced by Annexin V/PI staining, and is less pronounced in normal cells than in cancer cells [12].

In contrast, berberine has been widely reported to exert cytotoxic effects in various cancer cell lines. In HepG2 cells, berberine exhibited IC_50_ values of approximately 92–118 μM [35]. In colorectal cancer models (HCT‐116, SW‐480, and HT‐29), berberine achieved IC_50_ values of 34.6, 44.3, and 32.1 μM, respectively [36]. Notably, in triple‐negative breast cancer cell lines, including HCC70, BT‐20, and MDA‐MB‐468, berberine demonstrated exceptional potency, with IC_50_ values as low as 0.19, 0.23, and 0.48 μM, respectively, while sparing normal breast epithelial cells (MCF‐10A) at these low concentrations. Collectively, these findings support a therapeutic window in which berberine is more harmful to cancer cells than normal cells [37].

Taken together, the combined use of in silico, in vitro, and in vivo approaches provides a more comprehensive understanding of the toxicity and safety profiles. Although in silico approaches are useful as initial screening tools for compound safety owing to their efficiency and speed, their results do not always reflect the complex biological conditions in living organisms. Factors such as distribution, metabolism, and excretion can influence the toxicity profile of a compound, necessitating further validation through in vivo testing. Therefore, these three approaches should be used complementarily. In silico approaches provide initial estimates, in vitro assays confirm cytotoxic selectivity and underlying mechanisms, and in vivo studies ensure the accuracy of toxicity data in real biological contexts. Therefore, the integration of these approaches offers a more comprehensive understanding of the safety profile of the active compounds in *Fibraurea tinctoria* Lour.

### 4.3. Acute Oral Toxicity

Despite the many pharmacological activities of *Fibraurea tinctoria* Lour, there is a lack of information regarding its potential toxicity and adverse effects. This study evaluated the acute oral toxicity according to the OECD 425 guideline. Acute toxicity tests using experimental animals are needed to detect toxic effects that may occur shortly after the administration of a substance in a single or repeated dose within 24 h [38]. Toxicity studies have identified and classified the level of danger of a substance based on its lethality. This is important because, although natural products are widely used to treat various diseases and are often considered to have minimal or no side effects, several studies have shown that some bioactive compounds isolated from plants are toxic and may cause serious adverse effects in humans [39]. Therefore, this study provides useful information regarding the acute toxicity profile of *the Fibraurea tinctoria* Lour stem extract.

Several behavioral parameters were observed in this study, which showed no toxicological signs at either dose (2000 and 5000 mg/kg) compared to the control group. The results also showed that no animals died in the limit tests 2000 and 5000, indicating that the LD_50_ of the ethanol extract of *Fibraurea tinctoria* Lour was greater than 5000 mg/kg. According to the Globally Harmonized Classification System for Chemical Substances and Mixtures, the extract was categorized into group 5 (LD_50_ > 2000 mg/kg), implying that the extract has relatively low acute toxicity and is considered safe [40].

Observation of body weight parameters is a simple but sensitive toxicity index. Changes in body weight indicate the presence of side effects of a substance and are considered significant if the decrease is 10% of the initial body weight [41]. In addition to body weight parameters, absolute and relative organ weights are useful for assessing the potential toxicity of a compound, as they can indicate organ enlargement or shrinkage. Although these measurements cannot be used as absolute standards to determine organ damage or functional impairment, relative organ weight is considered a more sensitive marker of organ toxicity than body weight. The liver, kidneys, heart, lungs, and spleen are vital organs frequently affected or disrupted by toxic substances. Therefore, a thorough examination of these organs is necessary to identify signs of potential toxicity targeting the organs [42].

In this study, the administration of *Fibraurea tinctoria* Lour extract did not cause a reduction in body weight on Day 7 compared to that on Day 1 in any group. However, the measurement results on Day 14 showed a decrease in body weight of 7.46% in the 2000 mg/kg group, although it was still below the significance threshold (10%). Therefore, these results cannot be directly interpreted as indicative of toxicity. Nevertheless, this result is noteworthy because it could be an early indication of the effects of the extract on the body, particularly with long‐term use. Regarding relative organ weight, no significant differences were observed in the liver, kidneys, and spleen, whereas significant differences were observed in the heart and lungs when compared with the controls. Although changes in relative organ weight do not always directly correlate with structural damage or impaired organ function, this finding emphasizes the need for further histopathological and biochemical analyses to confirm the presence of pathological changes in the organs. Overall, the ethanol extract of *Fibraurea tinctoria* Lour is relatively safe, based on acute toxicity tests. However, indications of changes in certain organs emphasize the importance of continuing subchronic and chronic toxicity studies accompanied by more in‐depth histopathological analysis to obtain a more comprehensive picture of safety, especially for long‐term use.

## 5. Conclusions

This study revealed that berberine, palmatine, and jatrorrhizine fulfill Lipinski’s and Veber’s rules, suggesting good drug‐likeness and the potential to serve as lead compounds for further research. However, discrepancies have been observed between in silico and in vivo toxicity assessments. Although in silico studies predicted that these three compounds are orally toxic, an in vivo acute toxicity study of *Fibraurea tinctoria* Lour extract demonstrated no toxicity or adverse effects, with an LD_50_ value exceeding 5000 mg/kg, suggesting that the extract exhibits relatively low acute toxicity and is considered safe. This divergence highlights the limitations of prediction models that cannot fully capture the complex biological processes. Nevertheless, further in vivo studies, particularly in subchronic and chronic models, are required to clarify its long‐term safety and therapeutic viability.

## Conflicts of Interest

The authors declare no conflicts of interest.

## Funding

The authors received no specific funding for this work. The authors acknowledge the support provided by Universitas Padjadjaran for research facilities and express their gratitude to the Indonesia Endowment Fund for Education or Lembaga Pengelola Dana Pendidikan (LPDP), Indonesia (202108211607388), and the Academic Leadership Grant (ALG) Prof. Dr. Iman Permana Maksum, S.Si., M.Si. (1469/UN6.3.1/PT.00/2024).

## Data Availability

The datasets used in this study are available upon reasonable request to the corresponding authors.
